# Lifestyle and metabolic factors for nonalcoholic fatty liver disease: Mendelian randomization study

**DOI:** 10.1007/s10654-022-00868-3

**Published:** 2022-04-30

**Authors:** Shuai Yuan, Jie Chen, Xue Li, Rongrong Fan, Benoit Arsenault, Dipender Gill, Edward L. Giovannucci, Ju-sheng Zheng, Susanna C. Larsson

**Affiliations:** 1 Unit of Cardiovascular and Nutritional Epidemiology, Institute of Environmental Medicine, Karolinska Institutet, Nobelsväg 13, 17177 Stockholm, Sweden; 2 Centre for Global Health, Zhejiang University School of Medicine, Hangzhou, China; 3 Department of Gastroenterology, The Third Xiangya Hospital, Central South University, Changsha, China; 4 School of Public Health and the Second Affiliated Hospital, Zhejiang University School of Medicine, Hangzhou, China; 5 Centre for Global Health, Usher Institute, University of Edinburgh, Edinburgh, UK; 6 Department of Biosciences and Nutrition, Karolinska Institutet, Huddinge, Sweden; 7 Centre de Recherche de l’Institut Universitaire de Cardiologie et de Pneumologie de Québec, Quebec City, QC Canada; 8 Department of Medicine, Faculty of Medicine, Université Laval, Quebec, QC Canada; 9 Department of Epidemiology and Biostatistics, School of Public Health, Imperial College London, London, UK; 10 Clinical Pharmacology and Therapeutics Section, Institute for Infection and Immunity, St George’s, University of London, London, UK; 11 Clinical Pharmacology Group, Pharmacy and Medicines Directorate, St George’s University Hospitals NHS Foundation Trust, London, UK; 12 Novo Nordisk Research Centre Oxford, Old Road Campus, Oxford, UK; 13 Department of Epidemiology, Harvard T H Chan School of Public Health, Boston, MA USA; 14 Department of Nutrition, Harvard T H Chan School of Public Health, Boston, MA USA; 15 Key Laboratory of Growth Regulation and Translational Research of Zhejiang Province, School of Life Sciences, Westlake University, Hangzhou, China; 16 Department of Surgical Sciences, Uppsala University, Uppsala, Sweden

**Keywords:** Lifestyle, Mendelian randomization, Metabolic factor, Nonalcoholic fatty liver disease

## Abstract

The risk factors for nonalcoholic fatty liver disease (NAFLD) have not been clearly identified. We conducted a Mendelian randomization (MR) study to explore this. Independent genetic variants strongly associated with 5 lifestyle and 9 metabolic factors were selected as instrumental variables from corresponding genome-wide association studies (GWASs). Summary-level data for NAFLD were obtained from a GWAS meta-analysis of 8434 cases and 770,180 non-cases (discovery dataset) and another GWAS meta-analysis of 1483 cases and 17,781 non-cases (replication dataset). Univariable and multivariable MR analyses were performed. There were associations with NAFLD for lifetime smoking index (odds ratio (OR) 1.59, 95% confidence interval (CI) 1.31–1.93 per SD-increase), body mass index (BMI, OR 1.33, 95% CI 1.23–1.43 per SD-increase), waist circumference (OR 1.82; 95% CI 1.48–2.24 per SD-increase), type 2 diabetes (OR 1.21, 95% CI 1.15–1.27 per unit increase in log-transformed odds), systolic blood pressure (OR 1.17; 95% CI 1.07–1.26 per 10 mmHg increase), high-density lipoprotein cholesterol (OR 0.84, 95% CI 0.77–0.90 per SD-increase), and triglycerides (OR 1.23, 95% CI 1.15–1.33 per SD-increase). The associations for type 2 diabetes, systolic blood pressure, triglycerides, but not for high-density lipoprotein cholesterol remained strong after adjusting for genetically-predicted BMI. Genetic liability to type 2 diabetes mediated 51.4% (95% CI 13.4–89.3%) of the BMI-effects on NAFLD risk. There were suggestive inverse associations of genetically-predicted alcohol, coffee, and caffeine consumption, and vigorous physical activity with NAFLD risk. This study identified several lifestyle and metabolic factors that may be causally implicated in NAFLD.

**Supplementary Information:**

The online version contains supplementary material available at 10.1007/s10654-022-00868-3.

## Introduction

Nonalcoholic fatty liver disease (NAFLD) has become the most common form of chronic liver disease afflicting ~ 25% of the global population [1]. NAFLD has merged as the second leading indication for liver transplantation in the United States [2]. Due to obesity and diabetes epidemics, the disease burden of NAFLD is projected to increase 2 to threefold in Western countries as well as in several Asian areas by 2030 [3]. Although obesity is an important risk factor for NAFLD development, non-obese NAFLD patients have been identified as a large cluster making up ~ 20% of worldwide NAFLD population [4], which implies the etiological complexity of this disease as well as the possibility of prevention strategies targeting at other modifiable factors. Previous observational studies have identified modifiable factors for NAFLD, including metabolic traits, smoking, alcohol drinking, coffee consumption, and physical activity [5–7]. However, certain modifiable exposures, such as cigarette smoking [8] and alcohol drinking [9], have been inconsistently associated with NAFLD risk. The mutual relationship between metabolic syndrome and NAFLD are intertwined, especially in cross-sectional and case–control studies [10]. In addition, whether the associations of above factors with NAFLD risk are causal remains undermined due to potential residual confounding and reverse causality issues in observational studies.

Utilizing genetic variants as instrumental variables, Mendelian randomization (MR) is an epidemiological technique aimed at strengthening causal inference [11]. The approach carries two merits of minimizing confounding and diminishing reverse causality because genetic variants are randomly allocated at conception (thus unrelated to self-adopted and environmental factors) and cannot be modified by the development and progression of the disease [11]. Here, we conducted an MR study to investigate the associations of metabolic and lifestyle factors with risk of NAFLD. We also examined the obesity-independent effects of metabolic features on NAFLD as well as explored the mediators in the association between obesity and NAFLD.

## Methods

### Study design

**Fig. 1:**
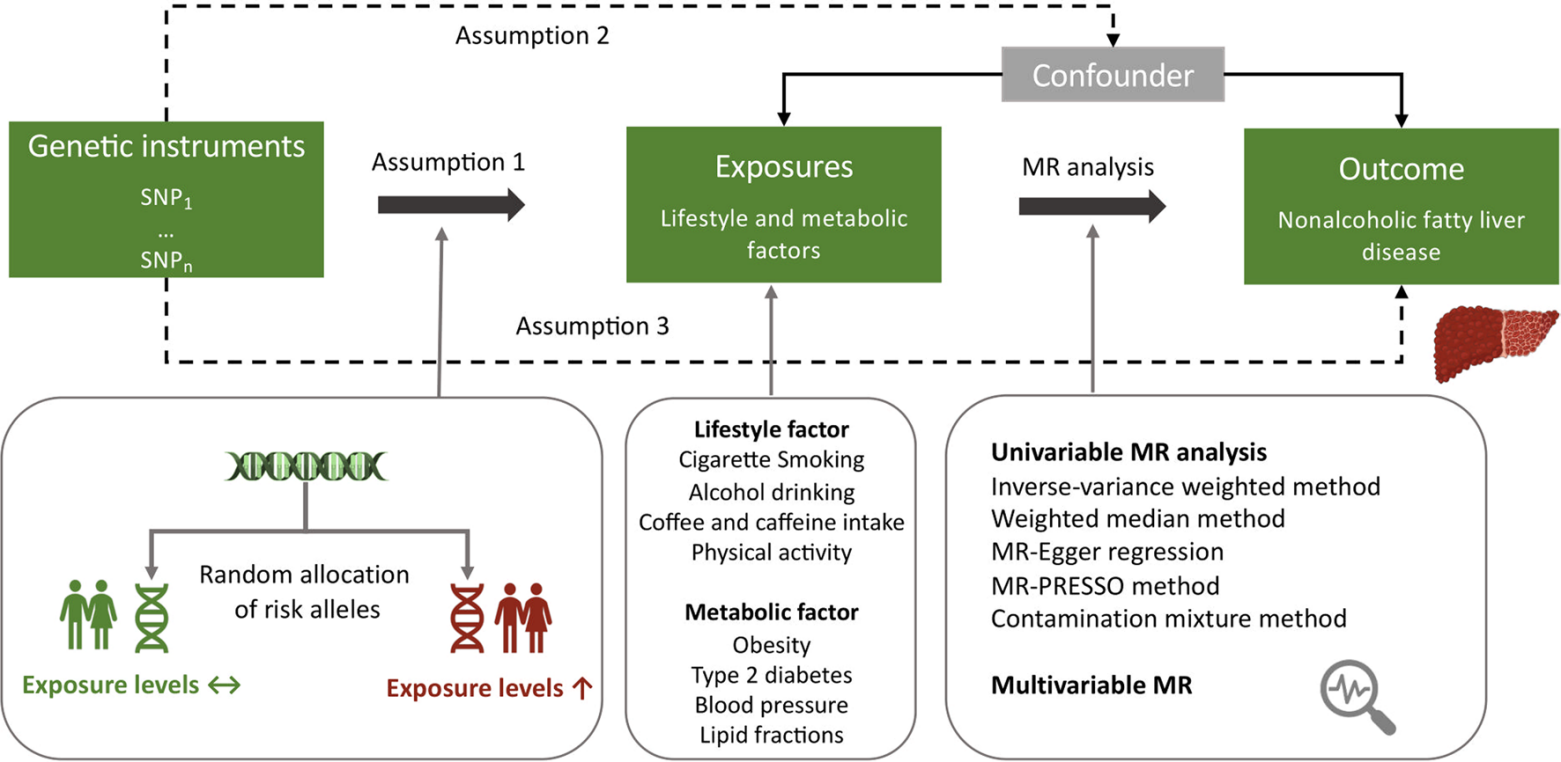
Study design overview

Figure 1 shows the study design. The present MR study included 14 modifiable factors (5 lifestyle and 9 metabolic factors). We firstly examined the associations of these factors with NAFLD in a large discovery dataset and then performed a replication analysis in an independent population. To increase power of the analysis, we combined estimates from two data sources. Multivariable MR method and mediation analysis were used. The analysis was conducted using summary-level data from published genome-wide association studies (GWASs) and the analytic process was in accordance with the STROBE-MR guidelines [12]. All studies included in cited GWASs had been approved by a relevant review board and all participants had provided the consent forms. The present MR analyses were approved by the Swedish Ethical Review Authority (2019‐02793).

### Genetic instrument selection

Single-nucleotide polymorphisms (SNPs) associated with 14 modifiable factors at the genome-wide significance level (*p* ≤ 5 × 10^–8^) were obtained from corresponding GWASs (Table 1). We estimated linkage disequilibrium among these SNPs based on the 1000 Genomes European reference panel [13]. SNPs in linkage disequilibrium (*r*^2^ ≥ 0.01) were excluded and the SNP with the smallest *p* value for the genome-wide association was attained. Genetic instrument selection for multivariable MR analysis followed the same criteria. For smoking behaviors, two sets of instruments (SNPs for smoking initiation and for lifestyle smoking index) were used for validation. Detailed information on GWASs of studied exposures, including number of participants and adjusted covariates, is presented in Table 1.

**Table 1:** Detailed information on used studies

Exposure or outcome	Unit	Participants included in analysis	Adjustments	IVs	PubMed ID
Lifestyle factor					
Smoking initiation	SD in prevalence of smoking initiation	1 232 091 European-descent individuals	Age, sex, and the first ten genetic principal components	311	30,643,251
Lifetime smoking index	SD change of lifetime smoking index	462 690 European-descent individuals	Genotyping chip and sex	126	31,689,377
Alcohol drinking	SD increase of log-transformed alcoholic drinks/week	941 280 European-descent individuals	Age, sex, and the first ten genetic principal components	84	30,643,251
Coffee consumption	50% change	375 833 European-descent individuals	Age, sex, body mass index, total energy, proportion of typical food intake, and 20 genetic principal components	12	31,046,077
Caffeine consumption	80 mg increase (equivalent to dose from 1 cup of coffee)	9876 European-descent individuals	Age, sex, study-site, fasting status, smoking status, and genetic principal components	2	27,702,941
Vigorous physical activity	≥ 3 versus 0 day/week	98 060 cases and 162 995 controls of European descent	Age, sex, genotyping chip, first ten genomic principal components, and center	5	29,899,525
Metabolic factor					
Body mass index	SD	806 834 European-descent individuals	Age, sex, and genetic 1–5 principal components	312	30,239,722
Waist circumference	SD	224 459 European-descent individuals	Age and study-specific covariates	44	25,673,412
Type 2 diabetes	One-unit in log-transformed odds	228 499 type 2 diabetes cases and 1 178 783 non-cases of multi-ancestries	Age, sex, and the first ten genetic principal components	497	32,541,925
Systolic blood pressure	10 mm Hg	Up to 1 006 863 European-descent individuals	Age, sex, BMI, genotyping chips	228	30,224,653
High-density lipoprotein cholesterol	SD	403 943 European-descent individuals	Age, sex, and genotyping chips	473	32,203,549
Apolipoprotein A-1	SD	393 193 European-descent individuals	Age, sex, and genotyping chips	383	32,203,549
Low-density lipoprotein cholesterol	SD	440 546 European-descent individuals	Age, sex, and genotyping chips	199	32,203,549
Triglycerides	SD	441 016 European-descent individuals	Age, sex, and genotyping chips	392	32,203,549
Apolipoprotein B	SD	439 214 European-descent individuals	Age, sex, and genotyping chips	225	32,203,549
Outcome					
NAFLD	Odds ratio	8434 NAFLD cases and 770 180 non-cases of European ancestry	Age, sex, and genetic principal components	–	34,841,290
NAFLD	Odds ratio	1483 NAFLD cases and 17 781 non-cases of European ancestry	Top 5 genetic principal components	–	32,298,765

*ID* identifier, *IV* instrumental variables, *NAFLD* nonalcoholic fatty liver disease, *SD* standard deviation

### Data sources for NAFLD

Summary-level data (i.e., beta coefficient and corresponding standard error) for the associations of exposure-associated SNPs with NAFLD were extracted from a GWAS meta-analysis including 8434 NAFLD cases and 770,180 non-cases (discovery stage) [14] and another GWAS including 1483 NAFLD cases and 17,781 non-cases (replication stage) [15] (Supplementary Table 1). Four GWASs (the Electronic Medical Records and Genomics, UK Biobank, FinnGen, and Estonian Biobank) were included in the discovery dataset [14]. The replication GWAS comprised data from 11 leading European tertiary liver centers [15]. Case definition and exclusion criteria in included NAFLD GWASs are shown in Supplementary Table 1. Detailed information on quality control refers to the cited GWAS papers [14, 15].

### Statistical analysis

SNPs in the exposure and outcome datasets were harmonized by coded and reference alleles to omit ambiguous SNPs with non-concordant alleles. We defined palindromic SNPs with ambiguous minor allele frequency > 0.45 and < 0.55 and all possible palindromic SNPs were excluded in a sensitivity analysis. A few missing instruments were not replaced by proxy SNPs given that a small proportion of missing generates limited influences on the results.

The inverse variance weighted (IVW) method was used as the main statistical analysis method and supplemented by four sensitivity analyses, including the weighted median [16], MR-Egger [17], MR-PRESSO [18], and contamination mixture [19] methods. The assumptions and strengths of used methods are summarized in Supplementary Table 2. Given previously identified shared genetic risk between obesity and metabolic traits [20], we used multivariable MR analysis with adjustment for genetically predicted BMI to assess the independent effects of metabolic traits on NAFLD. Likewise, we performed multivariable MR analysis to disentangle the effects of different blood lipids fractions on NAFLD. The analysis of mediation of metabolic traits on the association between obesity and NAFLD was performed under network MR framework where multivariable MR analysis was applied to adjust for the genetic association of the instruments with BMI. The mediation effect was calculated using the formula: (total effect—direct effect)/total effect and standard error of the mediation estimate was calculated using the propagation of error method [21]. Reverse MR analyses were performed for type 2 diabetes, BMI, and blood lipids based on 6 SNPs as genetic instruments for NAFLD [22]. Cochran’s Q statistic was used to assess the heterogeneity of SNP-estimates in each MR association. The *p* value of intercept test from MR-Egger regression was used to assess the horizontal pleiotropy [17]. The association with the *p* value < 0.004 (0.05/14 exposures) were deemed a significant association, and the association with the *p* value ≥ 0.004 and ≤ 0.05 were regarded as a suggestive association. The *F* statistic was calculated to measure the strength of used instruments and power was estimated using an online tool [23]. All tests were two-sided and performed using the TwoSampleMR [24], MR-PRESSO [18] and MendelianRandomization [25] packages in the R software (version 4.0.2).

## Results

The *F* statistic for instruments and estimated power for all analyses are shown in Table 2. All *F* statistics for the overall instruments were over 10, indicating a good strength of used genetic instruments. The power was low in the analysis of alcohol, coffee, and caffeine consumption, but adequate for the other studied exposures.

**Table 2:** *F* statistic and power estimation

Class	Exposure	SNPs	Variance explained	OR at 80% power for discovery analysis		OR at 80% power for replication analysis		OR at 80% power for combined analysis		*F* statistic
Lifestyle	Smoking initiation	311	0.023	≥ 1.21	≤ 0.80	≥ 1.52	≤ 0.53	≥ 1.19	≤ 0.81	60
Lifestyle	Lifetime smoking index	126	NA	NA	NA	NA	NA	NA	NA	NA
Lifestyle	Alcohol drinking	84	0.003	≥ 1.55	≤ 0.45	≥ 2.45	≤ 0.01	≥ 1.52	≤ 0.49	29
Lifestyle	Coffee consumption	12	0.005	≥ 1.44	≤ 0.57	≥ 2.12	≤ 0.05	≥ 1.40	≤ 0.60	334
Lifestyle	Caffeine consumption	2	0.013	≥ 1.27	≤ 0.73	≥ 0.38	≤ 0.73	≥ 1.25	≤ 0.75	5254
Lifestyle	Vigorous physical activity	5	NA	NA	NA	NA	NA	NA	NA	NA
Metabolic	Body mass index	312	0.027	≥ 1.19	≤ 0.81	≥ 1.48	≤ 0.56	≥ 1.18	≤ 0.82	71
Metabolic	Waist circumference	44	0.012	≥ 1.28	≤ 0.72	≥ 1.72	≤ 0.35	≥ 1.26	≤ 0.74	220
Metabolic	Type 2 diabetes	497	NA	NA	NA	NA	NA	NA	NA	NA
Metabolic	Fasting insulin	91	0.036	≥ 1.16	≤ 0.84	≥ 1.41	≤ 0.62	≥ 1.15	≤ 0.85	327
Metabolic	Fasting glucose	58	0.005	≥ 1.43	≤ 0.57	≥ 2.12	≤ 0.05	≥ 1.40	≤ 0.60	69
Metabolic	Systolic blood pressure	228	0.050	≥ 1.14	≤ 0.86	≥ 1.35	≤ 0.67	≥ 1.13	≤ 0.87	184
Metabolic	High-density lipoprotein cholesterol	473	0.142	≥ 1.08	≤ 0.91	≥ 1.21	≤ 0.80	≥ 1.08	≤ 0.91	279
Metabolic	Apolipoprotein A-1	383	0.119	≥ 1.09	≤ 0.91	≥ 1.23	≤ 0.78	≥ 1.09	≤ 0.91	281
Metabolic	Low-density lipoprotein cholesterol	199	0.078	≥ 1.11	≤ 0.89	≥ 1.28	≤ 0.73	≥ 1.11	≤ 0.89	339
Metabolic	Triglycerides	392	0.105	≥ 1.10	≤ 0.90	≥ 1.24	≤ 0.77	≥ 1.10	≤ 0.90	239
Metabolic	Apolipoprotein B	225	0.082	≥ 1.11	≤ 0.89	≥ 1.27	≤ 0.74	≥ 1.11	≤ 0.89	317

*NA* not available, *OR* odds ratio

**Fig. 2:**
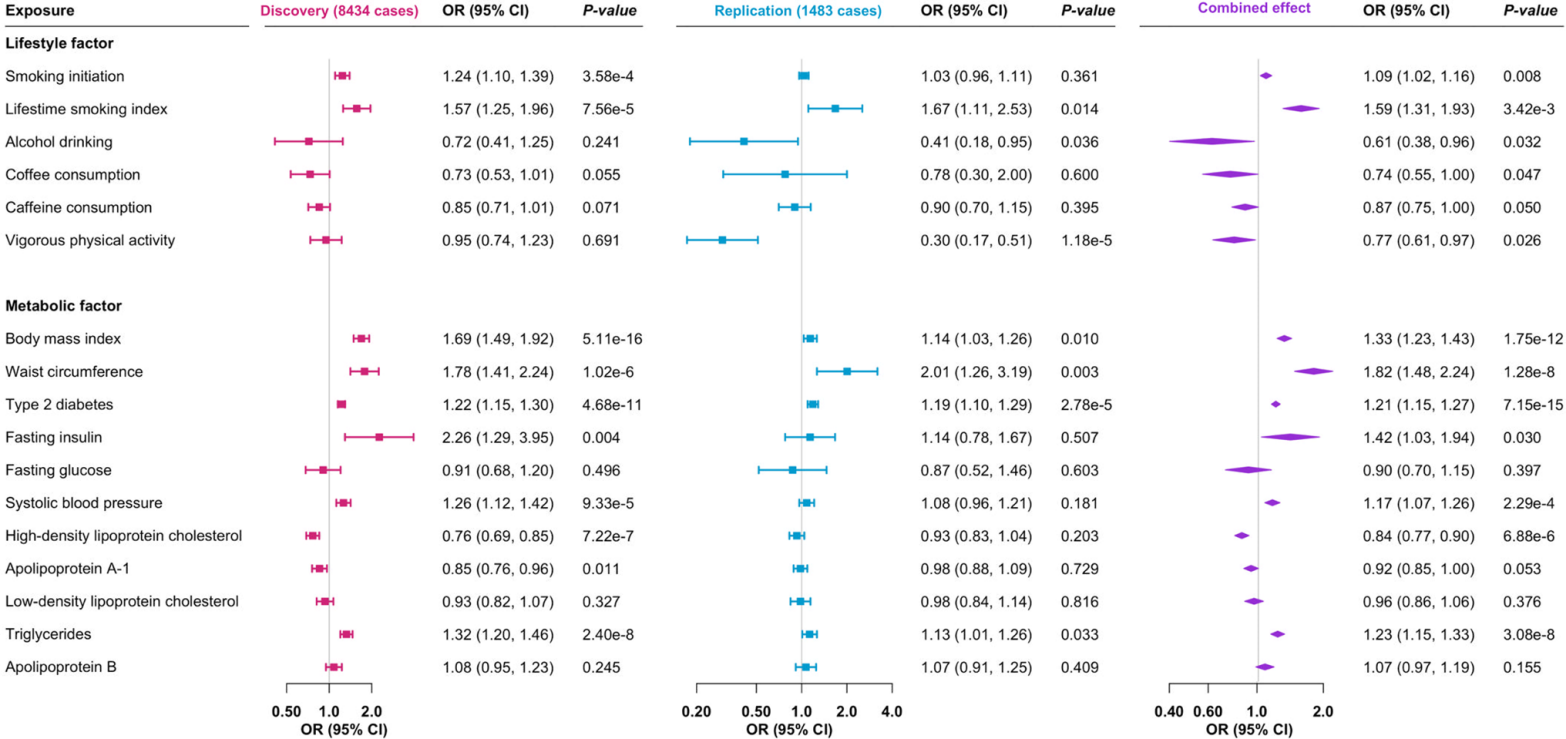
Associations of genetically predicted lifestyle and metabolic factors with risk of nonalcoholic fatty liver disease in discovery, replication, and combined datasets. *CI* confidence interval, *OR* odds ratio

Genetic predisposition to smoking was significantly associated with an increased risk of NAFLD in the discovery dataset and the association remained directionally consistent in the replication dataset (Fig. 1). The odds ratios (ORs) of NAFLD were 1.09 (95% confidence interval (CI) 1.02, 1.16; *p* = 0.008) for genetically predicted one-standard deviation (SD) increase in prevalence of smoking initiation and 1.59 (95% CI 1.31, 1.93; *p* = 3.42 × 10^–3^) for genetically predicted one-SD change of lifetime smoking index in the combined analysis. Other studied lifestyle factors, including alcohol drinking, coffee and caffeine consumption, and vigorous physical activity, were suggestively inversely associated with the risk of NAFLD in the combined dataset (Fig. 2). The ORs of NAFLD were 0.61 (95% CI 0.38, 0.96; *p* = 0.032) for genetically predicted one-SD increase of log-transformed alcoholic drinks/week, 0.74 (95% CI 0.55, 1.00; *p* = 0.047) for genetically predicted 50% increase in coffee consumption, 0.87 (95% CI 0.75, 1.00; *p* = 0.050) for genetically predicted 80 mg increase in caffeine consumption, and 0.77 (95% CI 0.61, 0.97; *p* = 0.026) for genetic predisposition to vigorous physical activity.

Six out of nine metabolic factors were significantly associated with NAFLD risk in the combined dataset (Fig. 2). There were associations for BMI (OR 1.33, 95% CI 1.23, 1.43; *p* = 1.75 × 10^–12^ per genetically predicted one-SD increase), waist circumference (OR 1.82; 95% CI 1.48, 2.24; *p* = 1.28 × 10^–8^ per genetically predicted one-SD increase), type 2 diabetes (OR 1.21, 95% CI 1.15, 1.27; *p* = 7.15 × 10^–15^ per one-unit increase in log-transformed odds), systolic blood pressure (OR 1.17; 95% CI 1.07, 1.26; *p* = 2.29 × 10^–4^ per genetically predicted 10 mm Hg increase), high-density lipoprotein cholesterol (OR 0.84, 95% CI 0.77, 0.90; *p* = 6.88 × 10^–6^ per genetically predicted one-SD increase), and triglycerides (OR 1.23, 95% CI 1.15, 1.33; *p* = 3.08 × 10^–8^ per genetically predicted one-SD increase). In the reverse MR analysis, genetic liability to NAFLD was associated with lower levels of high-density lipoprotein cholesterol, but not associated with other blood lipids, BMI, or risk of type 2 diabetes (Supplementary Table 3). High heterogeneity was observed in these analyses.

The observed associations were overall consistent across sensitivity analyses and between discovery and replication datasets (Supplementary Table 4). Moderate-to-high heterogeneity was observed in the analyses for alcohol drinking, type 2 diabetes, and lipid traits (Table 3 and Supplementary Table 4). We detected possible pleiotropy in the analyses for lifetime smoking index, type 2 diabetes, low-density lipoprotein cholesterol in the discovery analysis and in the analysis for body mass index in the replication analysis (Table 3 and Supplementary Table 4). However, these associations remained consistent after removal of outlier variants in MR-PRESSO analysis (Table 3 and Supplementary Table 4).

**Table 3:** Heterogeneity and pleiotropy assessment

Stage	Exposure	IVs	Cochran's Q	Intercept	*P* for intercept	Outliers
Discovery	Lifestyle factor					
	Smoking initiation	311	345	− 0.003	0.524	0
	Lifetime smoking index	126	124	0.017	0.013	0
	Alcohol drinking	84	165	0.009	0.329	4
	Coffee consumption	12	19	− 0.009	0.552	0
	Caffeine consumption	2	0	–	–	–
	Vigorous physical activity	5	2	0.071	0.303	0
	Metabolic factor					
	Body mass index	312	342	0.001	0.720	0
	Waist circumference	44	69	− 0.002	0.847	0
	Type 2 diabetes	497	929	0.007	0.006	6
	Fasting insulin	58	128	0.005	0.639	2
	Fasting glucose	91	159	0.008	0.152	1
	Systolic blood pressure	228	188	0.001	0.907	0
	High-density lipoprotein cholesterol	473	798	− 0.003	0.167	7
	Apolipoprotein A-1	383	730	− 0.002	0.530	6
	Low-density lipoprotein cholesterol	199	396	0.008	0.029	7
	Triglycerides	392	583	0.004	0.065	4
	Apolipoprotein B	225	415	0.006	0.095	6
Replication	Lifestyle factor					
	Smoking initiation	311	333	− 0.003	0.590	0
	Lifetime smoking index	126	118	0.001	0.968	0
	Alcohol drinking	84	151	0.005	0.719	3
	Coffee consumption	12	60	− 0.012	0.871	1
	Caffeine consumption	2	2	–	–	–
	Vigorous physical activity	5	1	0.107	0.530	0
	Metabolic factor					
	Body mass index	312	416	− 0.004	0.045	0
	Waist circumference	44	74	− 0.018	0.355	0
	Type 2 diabetes	497	925	− 0.001	0.882	5
	Fasting insulin	58	112	0.043	0.033	1
	Fasting glucose	91	148	0.007	0.514	1
	Systolic blood pressure	228	268	− 0.006	0.493	2
	High-density lipoprotein cholesterol	473	829	− 0.001	0.831	4
	Apolipoprotein A-1	383	744	− 0.001	0.689	4
	Low-density lipoprotein cholesterol	199	337	0.006	0.151	6
	Triglycerides	392	685	0.003	0.178	8
	Apolipoprotein B	225	337	0.003	0.410	5

*IVs* instrumental variables

**Fig. 3:**
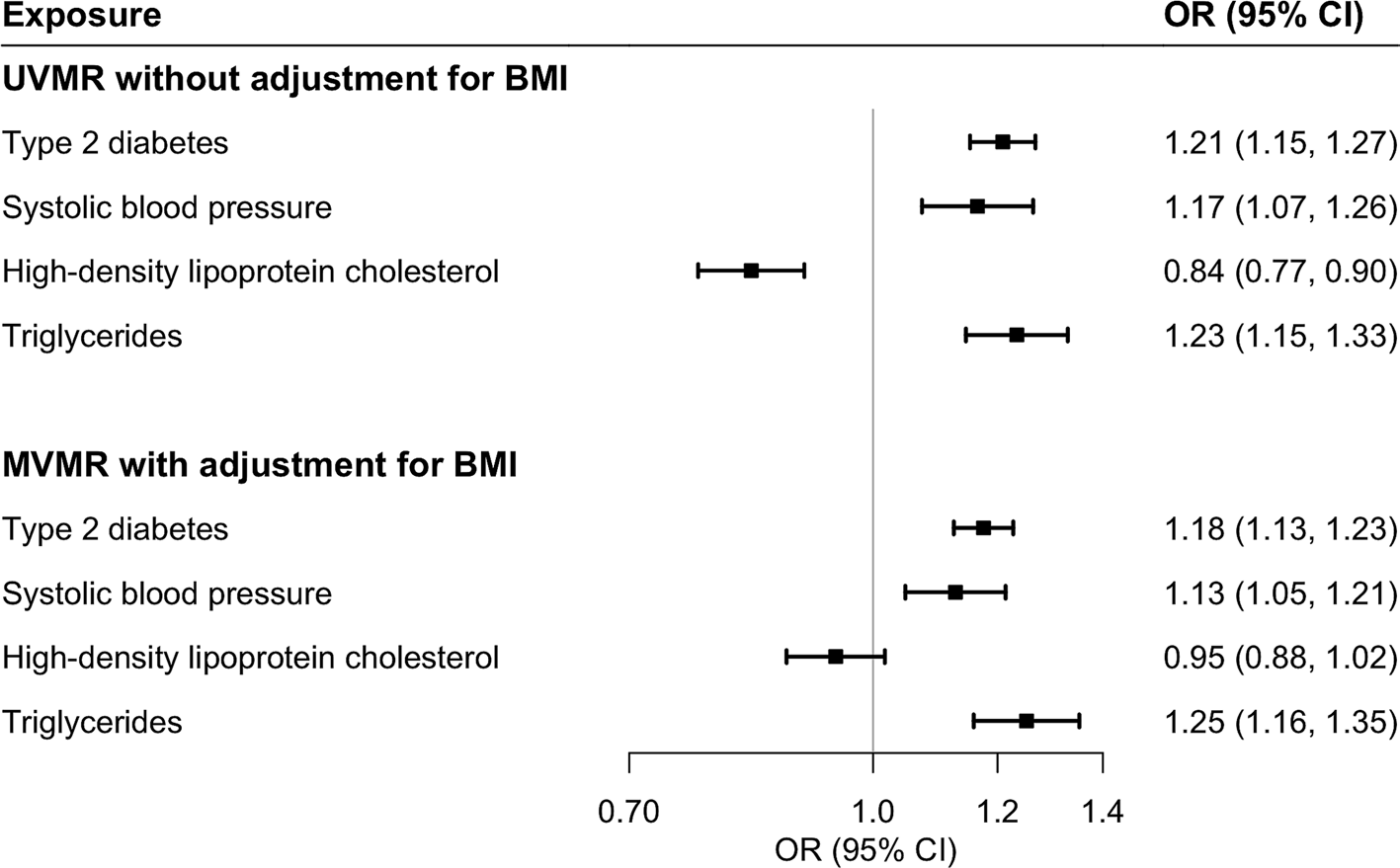
Genetically predicted BMI-adjusted associations of genetically predicted waist circumference, diabetes, systolic blood pressure, and lipids with risk of nonalcoholic fatty liver disease in combined dataset. *CI* confidence interval, *MVMR* multivariable Mendelian randomization, *OR* odds ratio, *UVMR* univariable Mendelian randomization

The associations for type 2 diabetes, systolic blood pressure, and triglycerides, but not for high-density lipoprotein cholesterol, remained similar in MR analysis with adjustment for genetically predicted BMI (Figs. 2 and 3). The inverse association for genetically predicted levels of high-density lipoprotein cholesterol and the positive association for genetically predicted levels of triglycerides became stronger in multivariable MR analyses with adjustment for genetically predicted levels of other related lipid traits (Supplementary Table 5). The association between BMI and NAFLD attenuated after adjusting for genetic liability to type 2 diabetes, and genetically predicted levels of high-density lipoprotein cholesterol and triglycerides (Table 4). Genetic liability to type 2 diabetes mediated 51.4% (95% CI 13.4%-89.3%) of BMI-effects on NAFLD risk (Table 4).

**Table 4:** Mediation of genetically predicted diabetes, systolic blood pressure, and lipids in the Mendelian randomization association between body mass index and nonalcoholic fatty liver disease

Model	Effect on NAFLD		Mediation effect	
	OR	95% CI	Mediation	95% CI
BMI without adjustment	1.33	1.23, 1.43	–	–
BMI adjusted for type 2 diabetes	1.15	1.04, 1.27	51.4%	13.4–89.3%
BMI adjusted for systolic blood pressure	1.33	1.23, 1.43	0.00%	− 39.8–37.8%
BMI adjusted for high− density lipoprotein cholesterol	1.20	1.09, 1.32	35.5%	− 2.5–73.5%
BMI adjusted for triglycerides	1.23	1.12, 1.34	27.7%	− 9.7–65.0%

*BMI* Body Mass Index, *CI* confidence interval, *NAFLD* nonalcoholic fatty liver disease, *OR* odds ratio

## Discussion

This MR study found that genetic predisposition to smoking, obesity, type 2 diabetes, high blood pressure, and dyslipidemia (low levels of low-density lipoprotein cholesterol and high levels of triglycerides) was associated with an increased risk of NAFLD. The associations for type 2 diabetes, systolic blood pressure, and triglycerides were independent of genetically predicted BMI. Low-density lipoprotein cholesterol and triglycerides appeared to be robust risk factors for NAFLD risk among lipid biomarkers. Type 2 diabetes mediated a large proportion of BMI-effects on NAFLD risk. There were suggestive inverse associations of genetically predicted moderate alcohol drinking, coffee and caffeine consumption, and vigorous physical activity with NAFLD risk.

An animal study found that four-week cigarette smoking worsened liver injury in obese rats with histological features of NAFLD via increased oxidative stress [26]. Observational studies of smoking and NAFLD have been inconsistent, with no association in a cross-sectional study with 90 NAFLD patients [8]. In another cross-sectional study including 2811 participants, individuals with one more pack of cigarette smoked per day had 1% higher risk of NAFLD [7]. This positive association was confirmed in a subsequent cohort study of 199,468 Korean adults where current smoking, pack-years, and urinary cotinine levels (a marker of tobacco smoke exposure) were positively associated with the risk of incident NAFLD [27]. Being in line with this cohort, our study found robust MR associations of smoking initiation and lifetime smoking index with NAFLD risk in two independent datasets, which strengthened the causal nature of this association. In addition, passive smoking in child and early adult lives has been associated with an increased later-life risk of developing NAFLD [28]. The underlying mechanisms behind the association between smoking and NAFLD may related to insulin resistance, hyperinsulinemia, dyslipidemia, hepatic steatosis, inflammation, and increased levels of catecholamine and glucagon, which can be induced by long-term smoking and nicotine use [27, 29].

Epidemiological evidence on the association between alcohol drinking and NAFLD risk is conflicting. A three-year longitudinal study with 4 waves’ repeated measurements including up to 3773 Japanese adults found that light to moderate alcohol consumption was associated with a reduced risk of NAFLD in both sexes [30]. This inverse association was also identified in a recent meta-analysis of 14 observational studies [31]. However, moderate alcohol use compared to nondrinking was associated with less improvement in steatosis and level of aspartate transaminase in 285 NAFLD patients after a mean follow-up period of 47 months [9]. An MR study including 266 NAFLD cases and 200 non-cases found that lifetime moderate alcohol consumption proxied by one genetic variant located in alcohol dehydrogenase (*ADH1B*) gene had no beneficial effects on NAFLD disease severity [32]. Nevertheless, animal data revealed that aldehyde dehydrogenase 2 deficiency ameliorated alcoholic fatty liver but worsened liver inflammation and fibrosis in mice [33]. Our MR analysis found a borderline inverse association between moderate alcohol drinking and NAFLD. Additional studies with a large sample size, the ability to assess the nonlinear associations, and with a comprehensive consideration of confounders, especially healthy lifestyle factors, will be needed to verify our findings and elaborate on underlying mechanisms.

Habitual coffee consumption has been consistently associated with a reduced risk of NAFLD and the association appears to be in a dose–response way in observational studies [34]. A recent MR study found no statistically significant association of coffee consumption instrumented by 4 SNPs (OR 0.76; 95% CI 0.51, 1.14) and 6 SNPs (OR 0.77; 95% CI 0.48, 1.25) with NAFLD risk using data from the UK Biobank with 1122 cases (one sub-dataset of our analysis) [35]. Compared with this study, our MR analysis with larger power (12 SNPs explaining larger variance in coffee consumption and ~ 9 times more cases) revealed a possible inverse association between coffee consumption and NAFLD risk. Besides, we detected a possible inverse association for caffeine consumption.

An inverse association between physical activity and NAFLD risk was observed in a meta-analysis including 6 cohort and 4 case–control studies [36] and subsequent studies [37, 38]. Compared with individuals with lowest physical activity levels, those with highest levels (similar to vigorous physical activity) had a lower odds of developing NAFLD (risk ratio, 0.79; 95% CI 0.71, 0.89) [36]. This association was in line with our MR finding. Future well-powered MR analysis is warranted to verify our finding.

Metabolic disorders, like obesity [39], type 2 diabetes [10], increased systolic blood pressure [10], and dyslipidemia [40], have been associated with NAFLD in observational studies. However, whether these metabolic traits are causally associated with NAFLD risk is unknown given that most associations were based on observational data. A previous MR study with 1122 NAFLD cases found harmful causal effects of overall and central obesity (represented by BMI and waist-to-hip ratio adjusted for BMI, respectively), type 2 diabetes, and triglycerides levels on NAFLD [41]. These associations were replicated in this updated MR study with more cases. Notably, increased systolic blood pressure and decreased levels of low-density lipoprotein cholesterol were identified as two new causal risk factors for NAFLD in our analysis. Even though we had limited evidence in support of the reverse impact of having NAFLD on type 2 diabetes, BMI, and blood lipids except for high-density lipoprotein cholesterol, which is not in line with observational studies [42], these null findings should be cautiously interpreted given high heterogeneity in these analyses as well as a few genetic instruments for NAFLD [22]. Thus, the effects of NAFLD on metabolic profiles need to be further explored.

Metabolic traits are usually tightly correlated with overweight and obesity. Estimating BMI-independent effect of these metabolic traits is of clinical importance especially in identifying high-risk group especially given a large number of lean NAFLD patients [4]. By subtracting effects of BMI, our multivariable MR analysis found independent roles of type 2 diabetes, elevated systolic blood pressure, and increased levels of triglycerides in the development of NAFLD. These findings are in line with associations observed in the normal weight population [43] and meanwhile convey the information that it may be beneficial to promote NAFLD screening as well as lifestyle intervention among individuals with an abnormal profile of glycemic traits, blood pressure or triglycerides. We also performed multivariable MR analysis among lipid fractions to pinpoint the robust lipid biomarker associated with NAFLD [44]. We found that low-density lipoprotein cholesterol and triglycerides appeared to be better indicators compared to other studied lipid traits. Additionally, mediation analysis found that type 2 diabetes and dyslipidemia might be intermediators in the pathway from BMI to NAFLD. The finding highlights that a good management of glucose and lipids levels in obese individuals may be an effective way to somehow neutralize the detrimental effects of overweight and obesity on NAFLD.

MR analysis has three important assumptions [11]. First, the selected instrumental variables should be strongly associated with the exposure of interest (assumption 1, relevance). In the present study, we selected SNPs that were associated with the exposures at the genome-wide significance level (*P* < 5 × 10^–8^) as instrumental variables from genome-wide association studies with large sample sizes. Second, used instrumental variables should not be associated with any confounders in the association between the studied exposure and outcome (assumption 2, independence). Given the study was based on summary-level data, a thorough examination of the associations between exposures and possible confounders was not possible. However, these instruments were widely used in previous MR studies on metabolic diseases [45, 46]. Third, the genetic instruments should influence the outcome only via the exposure, not via other alternative pathways (assumption 3, exclusion restriction). Although we could not completely rule out the possibility that our findings might be biased by horizontal pleiotropy, our results remained consistent across several sensitivity analyses and the MR-Egger and MR-PRESSO analyses detected limited evidence in support of strong pleiotropic effects.

There are several strengths of this MR study. The major merit is MR design which can minimize confounding and reverse causality to a large extent [11]. We explored the associations in two independent datasets to examine the consistency and then combined the associations from two data sources to increase the number of cases. Together with more SNPs used as instrumental variables compared to previous MR studies [32, 35, 41], our established associations should be better powered even though we might still have overlooked weak associations. The results remained overall consistent across several sensitivity analyses. In addition, we confined our analysis to the population of European descent, which effectively reduce the bias caused by the population structure bias. However, this study population of consistent ancestry may limit the generalizability of our findings to other populations.

Limitations need consideration when interpreting our results. The major issue for any MR study is horizontal pleiotropy that means selected genetic instrument variables influence the risk of outcome not via the exposure but other alternative pathways. However, this pleiotropic effect should not bias our results for two reasons. First, we detected limited evidence on pleiotropy from MR-Egger intercept test for most associations in the present MR analysis. For certain associations with significant indication of horizontal pleiotropy, there were few outliers detected by MR-PRESSO analysis and the association remained consistent or became even stronger after removal of outlying SNPs. Second, we performed multivariable MR for traits with strong phenotypic and genetic correlations and the associations remained stable after adjustment. Sample overlap between the exposure and outcome data sources might exist and thus bias our causal estimates towards observational associations by inflating the weak instrument bias [47]. Nonetheless, our SNPs were selected at the genome-wide threshold (strongly associated with the exposure) and all estimated F statistics were over 10, which indicates that the bias introduced by partial sample overlap should be minimal. Associations for certain exposures differed between the discovery and replication datasets, which might be caused population differences in certain features, like prevalence of obesity and vigorous physical activity. In addition, differences in NAFLD definition might cause heterogeneity in meta-analysis of associations across used data sources. For certain exposures, like alcohol consumption, the nonlinear association could not be estimated in the present MR analysis based on summary-level genetic statistics. Likewise, the gene-environmental interaction could not be assessed in summary-level data. The prevalence of different lifestyle and metabolic factors as well as NAFLD differs by age or sex [1, 48]. Whether the observed associations differ by age and sex could not be examined in the current MR study based on summary-level data and needs further investigation.

In conclusion, the present study provides MR data in support of causal roles of smoking, obesity, high systolic blood pressure, and dyslipidemia featured by low levels of low-density lipoprotein cholesterol and high levels of triglycerides, in the development of NAFLD. Mediation effects of type 2 diabetes and dyslipidemia in the association between BMI and NAFLD suggest the important role of the management of blood glucose and lipids in obesity in NAFLD prevention. The inverse associations for moderate alcohol drinking, coffee and caffeine consumption, and vigorous physical activity need confirmation in well-powered studies.

## Supplementary Information

Below is the link to the electronic supplementary material.

Supplementary file1 (XLSX 27 kb)

## References

[1] Younossi ZM, Koenig AB, Abdelatif D, Fazel Y, Henry L, Wymer M (2016). Global epidemiology of nonalcoholic fatty liver disease-Meta-analytic assessment of prevalence, incidence, and outcomes. Hepatology.

[2] Kim WR, Lake JR, Smith JM (2018). OPTN/SRTR 2016 annual data report: liver. Am J Transplant.

[3] Estes C, Anstee QM, Arias-Loste MT (2018). Modeling NAFLD disease burden in China, France, Germany, Italy, Japan, Spain, United Kingdom, and United States for the period 2016–2030. J Hepatol.

[4] Ye Q, Zou B, Yeo YH (2020). Global prevalence, incidence, and outcomes of non-obese or lean non-alcoholic fatty liver disease: a systematic review and meta-analysis. Lancet Gastroenterol Hepatol..

[5] Cotter TG, Rinella M (2020). Nonalcoholic fatty liver disease 2020: the state of the disease. Gastroenterology.

[6] Brunt EM, Wong VW, Nobili V (2015). Nonalcoholic fatty liver disease. Nat Rev Dis Primers.

[7] Koehler EM, Schouten JN, Hansen BE (2012). Prevalence and risk factors of non-alcoholic fatty liver disease in the elderly: results from the Rotterdam study. J Hepatol.

[8] Yilmaz Y, Yonal O, Kurt R, Avsar E (2010). Cigarette smoking is not associated with specific histological features or severity of nonalcoholic fatty liver disease. Hepatology.

[9] Ajmera V, Belt P, Wilson LA (2018). Among patients with nonalcoholic fatty liver disease, modest alcohol use is associated with less improvement in histologic steatosis and steatohepatitis. Clin Gastroenterol Hepatol.

[10] Lonardo A, Nascimbeni F, Mantovani A, Targher G (2018). Hypertension, diabetes, atherosclerosis and NASH: cause or consequence?. J Hepatol.

[11] Burgess S, Thompson SG (2015). Mendelian randomization: methods for using genetic variants in causal estimation.

[12] Skrivankova VW, Richmond RC, Woolf BAR (2021). Strengthening the reporting of observational studies in epidemiology using Mendelian randomization: the STROBE-MR statement. JAMA.

[13] Clarke L, Zheng-Bradley X, Smith R (2012). The 1000 genomes project: data management and community access. Nat Methods.

[14] Ghodsian N, Abner E, Emdin CA (2021). Electronic health record-based genome-wide meta-analysis provides insights on the genetic architecture of non-alcoholic fatty liver disease. Cell Rep Med..

[15] Anstee QM, Darlay R, Cockell S (2020). Genome-wide association study of non-alcoholic fatty liver and steatohepatitis in a histologically characterised cohort. J Hepatol.

[16] Bowden J, Davey Smith G, Haycock PC, Burgess S (2016). Consistent estimation in Mendelian randomization with some invalid instruments using a weighted median estimator. Genet Epidemiol.

[17] Bowden J, Davey Smith G, Burgess S (2015). Mendelian randomization with invalid instruments: effect estimation and bias detection through Egger regression. Int J Epidemiol.

[18] Verbanck M, Chen CY, Neale B, Do R (2018). Detection of widespread horizontal pleiotropy in causal relationships inferred from Mendelian randomization between complex traits and diseases. Nat Genet.

[19] Burgess S, Foley CN, Allara E, Staley JR, Howson JMM (2020). A robust and efficient method for Mendelian randomization with hundreds of genetic variants. Nat Commun.

[20] Civelek M, Wu Y, Pan C (2017). Genetic regulation of adipose gene expression and cardio-metabolic traits. Am J Hum Genet.

[21] Gill D, Cameron AC, Burgess S (2021). Urate, blood pressure, and cardiovascular disease: evidence from mendelian randomization and meta-analysis of clinical trials. Hypertension.

[22] Yuan S, Larsson SC (2022). Inverse association between serum 25-hydroxyvitamin D and nonalcoholic fatty liver disease. Clin Gastroenterol Hepatol.

[23] Brion MJ, Shakhbazov K, Visscher PM (2013). Calculating statistical power in Mendelian randomization studies. Int J Epidemiol.

[24] Hemani G, Zheng J, Elsworth B (2018). The MR-Base platform supports systematic causal inference across the human phenome. Elife.

[25] Yavorska OO, Burgess S (2017). MendelianRandomization: an R package for performing Mendelian randomization analyses using summarized data. Int J Epidemiol.

[26] Azzalini L, Ferrer E, Ramalho LN (2010). Cigarette smoking exacerbates nonalcoholic fatty liver disease in obese rats. Hepatology.

[27] Jung HS, Chang Y, Kwon MJ (2019). Smoking and the risk of non-alcoholic fatty liver disease: a cohort study. Am J Gastroenterol.

[28] Wu F, Pahkala K, Juonala M (2021). Childhood and adulthood passive smoking and nonalcoholic fatty liver in midlife: a 31-year cohort study. Am J Gastroenterol.

[29] Malfertheiner P, Schütte K (2006). Smoking–a trigger for chronic inflammation and cancer development in the pancreas. Am J Gastroenterol.

[30] Moriya A, Iwasaki Y, Ohguchi S (2015). Roles of alcohol consumption in fatty liver: a longitudinal study. J Hepatol.

[31] Wongtrakul W, Niltwat S, Charatcharoenwitthaya P (2021). The effects of modest alcohol consumption on non-alcoholic fatty liver disease: a systematic review and meta-analysis. Front Med (Lausanne).

[32] Sookoian S, Flichman D, Castaño GO, Pirola CJ (2016). Mendelian randomisation suggests no beneficial effect of moderate alcohol consumption on the severity of nonalcoholic fatty liver disease. Aliment Pharmacol Ther.

[33] Kwon HJ, Won YS, Park O (2014). Aldehyde dehydrogenase 2 deficiency ameliorates alcoholic fatty liver but worsens liver inflammation and fibrosis in mice. Hepatology.

[34] Chen YP, Lu FB, Hu YB, Xu LM, Zheng MH, Hu ED (2019). A systematic review and a dose-response meta-analysis of coffee dose and nonalcoholic fatty liver disease. Clin Nutr.

[35] Zhang Y, Liu Z, Choudhury T, Cornelis MC, Liu W (2021). Habitual coffee intake and risk for nonalcoholic fatty liver disease: a two-sample Mendelian randomization study. Eur J Nutr.

[36] Qiu S, Cai X, Sun Z (2017). Association between physical activity and risk of nonalcoholic fatty liver disease: a meta-analysis. Therap Adv Gastroenterol..

[37] Vilar-Gomez E, Nephew LD, Vuppalanchi R (2021). High-quality diet, physical activity, and college education are associated with low risk of NAFLD among the US population. Hepatology.

[38] Li Y, He F, He Y (2019). Dose-response association between physical activity and non-alcoholic fatty liver disease: a case-control study in a Chinese population. BMJ Open.

[39] Li L, Liu DW, Yan HY, Wang ZY, Zhao SH, Wang B (2016). Obesity is an independent risk factor for non-alcoholic fatty liver disease: evidence from a meta-analysis of 21 cohort studies. Obes Rev.

[40] Deprince A, Haas JT, Staels B (2020). Dysregulated lipid metabolism links NAFLD to cardiovascular disease. Mol Metab.

[41] Liu Z, Zhang Y, Graham S (2020). Causal relationships between NAFLD, T2D and obesity have implications for disease subphenotyping. J Hepatol.

[42] Yki-Järvinen H (2014). Non-alcoholic fatty liver disease as a cause and a consequence of metabolic syndrome. Lancet Diabetes Endocrinol.

[43] Sookoian S, Pirola CJ (2017). Systematic review with meta-analysis: risk factors for non-alcoholic fatty liver disease suggest a shared altered metabolic and cardiovascular profile between lean and obese patients. Aliment Pharmacol Ther.

[44] Yuan S, Tang B, Zheng J, Larsson SC (2020). Circulating lipoprotein lipids, apolipoproteins and ischemic stroke. Ann Neurol.

[45] Yuan S, Larsson SC (2020). An atlas on risk factors for type 2 diabetes: a wide-angled Mendelian randomisation study. Diabetologia.

[46] van Oort S, Beulens JWJ, van Ballegooijen AJ, Handoko ML, Larsson SC (2020). Modifiable lifestyle factors and heart failure: a Mendelian randomization study. Am Heart J.

[47] Burgess S, Davies NM, Thompson SG (2016). Bias due to participant overlap in two-sample Mendelian randomization. Genet Epidemiol.

[48] Lonardo A, Nascimbeni F, Ballestri S (2019). Sex differences in nonalcoholic fatty liver disease: state of the art and identification of research gaps. Hepatology.

